# Predicting citations in Dutch case law with natural language processing

**DOI:** 10.1007/s10506-023-09368-5

**Published:** 2023-06-28

**Authors:** Iris Schepers, Masha Medvedeva, Michelle Bruijn, Martijn Wieling, Michel Vols

**Affiliations:** 1 Department of Legal Methods, Faculty of Law, University of Groningen, Groningen, The Netherlands; 2 Center for Language and Cognition Groningen, Faculty of Arts, University of Groningen, Groningen, The Netherlands; 3 Center for Law and Digital Technologies, Faculty of Law, University of Leiden, Leiden, The Netherlands

**Keywords:** Machine learning, Case law, Natural language processing, Citation analysis, Judicial decisions

## Abstract

With the ever-growing accessibility of case law online, it has become challenging to manually identify case law relevant to one’s legal issue. In the Netherlands, the planned increase in the online publication of case law is expected to exacerbate this challenge. In this paper, we tried to predict whether court decisions are cited by other courts or not after being published, thus in a way distinguishing between more and less authoritative cases. This type of system may be used to process the large amounts of available data by filtering out large quantities of non-authoritative decisions, thus helping legal practitioners and scholars to find relevant decisions more easily, and drastically reducing the time spent on preparation and analysis. For the Dutch Supreme Court, the match between our prediction and the actual data was relatively strong (with a Matthews Correlation Coefficient of 0.60). Our results were less successful for the Council of State and the district courts (MCC scores of 0.26 and 0.17, relatively). We also attempted to identify the most informative characteristics of a decision. We found that a completely explainable model, consisting only of handcrafted metadata features, performs almost as well as a less well-explainable system based on all text of the decision.

## Introduction

With the ever-growing accessibility of case law online, it has become (almost) impossible to analyse all case law manually. One country where this problem is becoming increasingly prevalent is the Netherlands. In the past years, the percentage of decisions published online (on rechtspraak.nl) has almost doubled, from 4.1% in 2017 to 7.8% in 2021.^1^. This currently amounts to over 41,000 decisions per year, ranging from lower level courts, such as district courts, to higher courts, such as the courts of appeal and the Supreme Court. Even though the current case law database consists of ‘only’ around 600,000 decisions, it is already challenging for legal practitioners and researchers to find the ones relevant to their case or research. The ambition of the Dutch council for the judiciary is to implement a system in which 75% of all decisions are published.^2^. If no measures are taken to improve the data’s searchability, this will lead to even more problems with retrieving the relevant decisions. The increase of legal data availability calls for ways to automatically analyse this data, since doing so manually is too time-consuming.

A way to automatically analyse large amounts of textual data is by using machine learning (ML). Over the past few decades, ML techniques have been used for various tasks in the field of artificial intelligence and law. For instance, in legal outcome forecasting (as defined by Medvedeva et al. 2022), the outcome of a court case is predicted from the facts (formulated before the outcome of a case was known) of the case with the help of classification algorithms and natural language processing (NLP) techniques. Research by e.g. Medvedeva et al. (2021b) shows that the text of legal proceedings holds valuable information for this task.

Our present study investigates whether it is possible to forecast if a decision of a Dutch court will be cited in a future Dutch court decision. We use Dutch court data as a case study, as this data is available online. Fowler and Jeon (2008) have shown that case authority, which is the extent to which a decision is deemed important for settling other legal disputes, and citations are related. Consequently, when forecasting incoming citations, one may thereby forecast the authority of a decision before this authority is even acknowledged in other case law (i.e. through actual citations).

Citing case law can have different functions. In cases with a common law system, such as the United Kingdom, the law is ‘judge-made’, meaning there is no written law. The law is created and developed through court decisions. In common law countries, judges decide along the lines of earlier decisions made in similar cases (i.e. precedents). As such, citations have a different function in these countries than in countries with a civil legal culture where most of the law is codified. The Netherlands follows the civil law tradition: the law is created by a legislator, and unlike common law countries, the Netherlands does not adopt the doctrine of *stare decisis*. Consequently, previous cases are taken into account in the Netherlands (especially from higher courts), but judges are not obliged to follow the legal precedents. As such, the authority of cases and the function of citations differ between common law and civil law countries. In fact, since civil law countries (such as the Netherlands) are not bound by historical precedents, and thus not obliged to refer to previous decisions, one might even argue that the relationship between citations and authority is even stronger in civil law countries than in common law countries (Zweigert and Kötz 1998).

Van Opijnen (2016) states that a system for tagging the importance of decisions is essential for the accessibility of legal big data. An example of this implementation can be found in HUDOC, the European Court of Human Rights (ECtHR) online database. In this database, it is possible to filter case law by their importance levels.^3^. These importance levels have been decided for and have been added manually to each case that has been uploaded, which makes it easy to implement an importance filter. However, in an existing database that has no previously recorded importance levels, such as the Dutch rechtspraak.nl, this is not possible. Therefore, our study aims to contribute to the first step in implementing an authority ranking system for Dutch case law.

Contrary to the ECtHR implementation, we do not distinguish between different importance levels. Rather, we differentiate between a clearly defined class encompassing all non-authoritative decisions (receiving zero incoming citations), and a sliding scale class encompassing all other decisions that may or not be authoritative (receiving any number of citations greater than zero). We perform a binary classification task in which we forecast whether or not a decision is cited by other case law at all, thereby predicting if the decision will be non-authoritative (meaning ‘uncited’) or not. This prediction could be used to help label decisions by filtering out the (likely) non-authoritative cases. This implementation will therefore not identify the most important decisions, but it helps filter out any decisions that are certainly not important, which is especially useful when navigating through the large amounts of available data. Therefore, this system can help legal practitioners to substantially reduce the time spent on preparation for their research or case.

Besides building a model that forecasts whether or not a court decision will be cited, we also aim to gain insight into the most informative features for determining citability. We investigate whether certain words, phrases, or characteristics increase the likelihood of a decision getting cited or remaining uncited. In doing so, we hope to contribute practically to implementing a ‘(non-)authority filter’ on rechtspraak.nl.

The following section discusses prior research related to prediction and forecasting tasks and network analysis using legal data. Section 3 describes the data and features used in our experiments. Next, in Sect. 4 we explain the methods and setup of the experiments that we have conducted. In Sect. 5 we report the results of these experiments. Finally, we discuss these results in Sects. 6 and 7 and draw conclusions.

## Background

Traditional research in the field of law usually consists of doctrinal analysis. Yet, in recent years, empirical methods have been used as well (Vols 2021a, b). In our work, we aim to forecast whether or not a case was cited to determine the (non-)importance of a decision by combining the knowledge gained from using legal citation analysis and machine learning techniques applied to legal data. Machine learning techniques have been used for a variety of tasks in the legal field. Some examples of these tasks are extracting and summarising the most important parts of cases (e.g., Moens et al. 1997; Pandya 2019), extracting semantic legal metadata from laws (e.g., Spinosa et al. 2009; Sleimi et al. 2018, 2021), detecting unfair clauses in terms and conditions (e.g., Lippi et al. 2019), identification of the subject of case law (e.g., Medvedeva et al. 2021a) and, as mentioned before, legal decision prediction. The latter has been a relatively common practice in the field of AI and law. It has been performed on legal data from, e.g., Chinese courts (Zhong et al. 2018), the UK Supreme Court (Strickson and De La Iglesia 2020), the French Supreme Court (Şulea et al. 2017a, b), the Supreme Court of the Philippines (Virtucio et al. 2018), the Supreme Court of the United States (Katz et al. 2017), and, most often, the European Court of Human Rights (e.g., Chalkidis et al. 2019; Medvedeva et al. 2020; Kaur and Bozic 2019; O’Sullivan and Beel 2019). An extensive overview of artificial intelligence techniques used in legal analytics can be found in Ashley (2017), and an overview of recent advances in the field is provided by Whalen (2020). A discussion of previous work about predicting court outcomes can be found in Medvedeva et al. (2022). This work indicates that legal big data suits numerous machine learning (ML) and natural language processing (NLP) techniques.

Another empirical research method that has been used in the field of law, is citation analysis. Networks can be found in any research area, including the nerve cells in the human brain, relations in society, web pages on the internet, and citations of scientific literature (Barabási and Bonabeau 2003). Researchers in numerous fields have found that many networks are not distributed randomly, but instead are commanded by a small number of nodes that make up the majority of the connections. These important nodes, also called ‘hubs’, sometimes have a seemingly unlimited number of connections that appears to have no scale. Barabási and Bonabeau (2003) state that it is important to determine if one is dealing with a scale-free network to properly understand its behaviours. In legal citation networks, we also find characteristics of a scale-free network. A legal citation network is formed by the connections between legal documents (the nodes) through citations (the edges). While a relatively small number of highly influential ‘landmark decisions’ attract a substantial number of connections, the majority of decisions do not receive any citation at all. This is supported by findings of Leitão et al. (2019), who investigated the citations over time of over 17,000 admitted cases from the European Court of Human Rights up until 2016. Both Barabási and Bonabeau (2003) and Leitão et al. (2019) state that scholars or practitioners are more likely to cite well-established or well-known documents when they cite previous sources. In the legal field, this reinforces the influence and connectivity of those landmark cases, which is also known as the rich-get-richer effect, or ‘preferential attachment’. As a result, highly cited cases become hubs within the legal citation network, shaping its structure and dynamics.

An extensive history of citation analysis in law can be found in Whalen (2016), in which different applications of network analysis on legal data are described. For instance, there has been research into the social networks of criminals, but there has also been work that views statutes, regulatory codes, or case law from a network analysis viewpoint. Leitão et al. (2019) perform an analysis of the evolution of precedents over time and attempt to explain the importance of decisions by means of the Bass model. They find that the major part of how decisions are cited can be explained by a combination of the rich-get-richer mechanism and external factors, in which the former tends to play a larger role. According to Fowler and Jeon (2008), it is possible to rank decisions of the Supreme Court of the United States on authority using citation network data. While citations can happen for different reasons, they unquestionably provide evidence for the use of a previous decision, thus making the number of incoming citations a useful quantitative measure of the usage of a decision within courts. They describe an *authority score*, which is based on the number of times a decision gets cited, and the quality of these citing decisions. They argue that this authority score is able to identify decisions that legal experts label as ‘landmark decisions’. Some benefits of their score are that it takes much less effort to calculate than to have an expert form an opinion and that there is no chance of a subjective bias, which a human expert might exhibit. The assigned scores even show which decisions might become important in the future. Kuppevelt and Dijck (2017) present a similar tool specifically developed for Dutch case law.

Sadl and Tarissan (2020) demonstrate the potential of using legal network analysis to study the Court of Justice of the European Union (CJEU). They are able to identify landmark decisions and crucial legal developments by using measures of centrality to reflect case importance. They detect the fluctuating importance of decisions by using complementary centrality measures, and argue that the relative in-degree score of a decision can provide a comprehensive view of the evolution of case importance. They address critiques of network analysis and conclude that it may never replace doctrinal analysis, but it can provide an objective, transparent basis for legal research. The work of Sartor et al. (2023) provides an automated extraction pipeline for CJEU case law. They present a valuable tool to create and analyse networks, and they argue that automating the process will support traditional legal research too. Derlén and Lindholm (2017) go one step beyond finding the most authoritative nodes in a network, and use several metrics on a CJEU network to determine the current precedential power of a decision to detect if it is still ‘good law’. They conclude that the metrics they use are not always compliant with the expert opinion of lawyers and that researchers should be mindful of the methods they use. As investigated by Derlén and Lindholm (2017), decisions can become redundant over time, but can also be ‘awakened’ after a while and suddenly start gathering citations years after their publication. These phenomena are called ‘Sleeping Beauties’ (Ke et al. 2015). Hernandez Serrano et al. (2020) presented an algorithm that aims to identify these decisions in CJEU case law. Their methodology is compliant with traditional network metrics, and they find that the most highly influential decisions in a network tend to go unnoticed for a longer amount of time than other decisions (almost 11 months longer).

Winkels and de Ruyter (2011) performed an analysis of case law of the Dutch Supreme Court. Their research shows that decisions cited most seem to ‘fill gaps in legislation’. This means that the decision made by the court is not covered by a piece of legislation yet, and the decision is cited often until the ‘gap’ is fixed. They also find that the most cited decisions are often about procedural law. Still, this observation may be influenced by the fact that they only analysed data from the Supreme Court. They compare their research to Fowler and Jeon (2008) and say that even though the Dutch Supreme Court cites fewer decisions than the US Supreme Court, the number of citations seems to be a good indicator of authority for Dutch case law as well. From the aforementioned studies we deduce that decisions which are not cited are less authoritative. By identifying these uncited decisions, it should be possible to filter out decisions that are less authoritative and, therefore, less interesting for legal practitioners.

Though the use of citation networks has been present in legal research, work on predicting the number of citations using machine learning has yet to be published. However, Mones et al. (2021) use a Random Forest classifier to predict links between decisions, which they find to be highly predictable. They argue that an empirical understanding of the application of legislation is essential as it not only supports equality in treatment, but also improves effectiveness and consistency. They find that the most informative factors to a prediction change over time: the content of a decision plays a smaller role over time, whereas features of the network itself grow more important to the prediction. Comparable to Sadl and Tarissan (2020), Mones et al. (2021) argue that algorithmically identifying relevant decisions could never fully replace the lawyer’s insights, but it can definitely provide useful advantages.

There is some work on the statistical ranking of Dutch decisions. Van Opijnen (2012) attempts to measure legal authority by doing an extensive citation network analysis using half a million Dutch decisions. He defines and measures legal authority in various ways, namely the number of incoming citations from other case law, the number of publications in legal journals, the number of annotations published with the decisions, and his own metric, the ‘Marc In-Degree’ (calculated as $1 + log_2(C)$, in which *C* is the number of incoming citations). The author concludes that exogenous variables (e.g., incoming citations) are relevant for determining case authority and that endogenous variables he examined (e.g., the type of court or the length of the decision) by themselves are not sufficient for determining reliable results. He then builds upon these findings by creating the MARC (‘Model for Automated Ranking of Case Law’) score (Van Opijnen 2013). This model is implemented in the internal database of the Dutch judiciary to calculate an authority score for each decision. The model consists of two parts: the first part of the model analyses the decisions that have not been cited yet (the ‘publication period’), and the second part analyses the decisions that have been cited (the ‘citation period’). The score is then constantly updated based on the changing incoming citations. The first part of the statistical model is based only on several selected (primarily) endogenous variables, which he concludes to be less trustworthy than exogenous variables in his previous work Van Opijnen (2012). However, Van Opijnen (2013) concludes that even though the endogenous predictors do not add much to a model that has access to the exogenous predictors, the endogenous predictors have enough predictive value on their own. We also evaluate several of these variables in our approach to predicting whether or not a case is cited.

In the present study, we are expanding upon prior research by Van Opijnen (2012) and Van Opijnen (2013) by assessing the (non-)authority of Dutch case law. We do this by predicting whether or not rechtspraak.nl decisions are cited. For this, we solely use endogenous features from the metadata and the texts of decisions (extracted through NLP techniques), all of which are available from the moment the decisions are published. In doing so, we also aim to determine if any endogenous variables, not described by Van Opijnen, are valuable to include in determining whether or not a case is cited. Our approach is, therefore, a first step towards determining the case’s authority, as cases which are not cited are also not authoritative.

## Data

### Data collection

The data used for this study consist of Dutch case law from rechtspraak.nl. The content and metadata of all published decisions can be downloaded in XML format via *Open Data van de Rechtspraak*, the Open Data of the Judiciary (ODR).^4^ The downloaded ODR dataset contains about 3,090,000 files from 1911 up to 2022, sorted per month. However, the contents of a large number of ODR files are not available to the public. Some are only available to the judiciary in a particular archive, and some publications have been revoked. These files were filtered out, thereby we use the oldest 60% of the data for our experiments

All published files containing decisions have a relatively consistent structure that can be found online in the technical documentation.^5^. The structure of the text of the decision itself varies slightly per court of law. Still, it usually contains an introduction, process flow, considerations, and a decision. There is, however, much variation in the aesthetic formatting, as there are likely many different editors working on these files, each using their own style conventions.

As the incoming and outgoing citations are not adequately registered in ODR, we used another governmental dataset for this, dubbed the *Linked Data Overheid*, ‘Linked Data Government’ (LIDO). This dataset contains all of the links between a large number of governmental web pages, which also include citations to case law. This dataset is updated monthly as well.^6^ The citations in this dataset were extracted from the text by a sophisticated algorithm, the LinkeXtractor (Van Opijnen 2018). This algorithm recognises various citation formats but may make mistakes in rare cases. For instance, a 1905 Supreme Court decision^7^ cites, according to the LinkeXtractor, the 2001 ECtHR decision *Van den Hoogen v. the Netherlands*,^8^ which is impossible. The extractor deduced this citation from the phrase *’van den Hoogen Raad‘* (which means ‘by the Supreme Court’ in old Dutch and matches part of the name of the 2001 case). We filtered out any citations to future case law to correct these erroneous citations. We have also filtered out citations due to ‘formal relations’, i.e. , a decision by a lower or higher court in the same case. We are only interested in citations that are made because of the relevance of the content of a decision, as only these citations indicate the authority of a decision. However, we include formal relations as a feature for predicting whether or not the decisions get cited, which we elaborate on later in this Section.

### Data selection

The Dutch Council for the Judiciary started publishing the data online in December 1999. We do not have access to outgoing citations from decisions that are not available online, so we chose to exclude decisions from before 1999 that have been published after their ruling date.

We focus on three types of courts: the district courts (DC), the Council of State (CS), and the Supreme Court (SC). The Supreme Court is the peak court level in private, criminal and tax cases, while the Council of State is the highest court for administrative law. In 2022, there are eleven district courts, which we combined, as they generally treat the same types of cases in first instance, and there are not enough decisions published for each court separately. Courts that were renamed or abolished in the past have also been included in this dataset. For example, there used to be one district court for the eastern part of the Netherlands, but it was later split into two district courts for the provinces of Overijssel and Gelderland. The three types of courts (DC, CS and SC) were distinguished from each other, as this allows us to compare citations regarding decisions at first instance and at their final appeal (SC/CS versus DC) and to compare between the area of law (SC versus CS). Our datasets contain decisions up to the 31st of August, 2022, which leaves us with 29,007 SC decisions, 59,356 CS decisions, and 153,735 DC decisions.

**Fig. 1:**
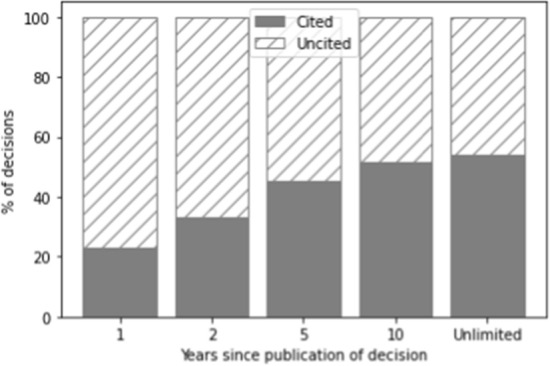
Distribution of cited vs. non-cited decisions for the Supreme Court over time

**Fig. 2:**
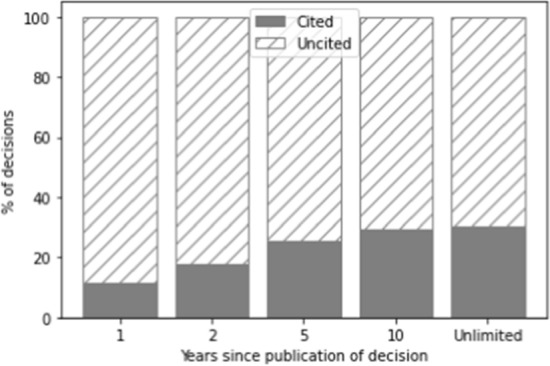
Distribution of cited vs. non-cited decisions for the Council of State over time

**Fig. 3:**
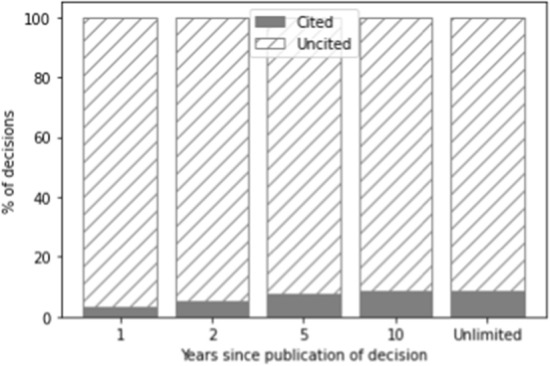
Distribution of cited vs. non-cited decisions for the district courts over time

The number of citations is determined in relation to a specific time span during which the decision was cited. In Figs. 1, 2, and 3, the grey part of each bar indicates decisions that have been cited within one year, two years, five years, ten years, and the entire period available, respectively, whereas the part of the bar with diagonal lines indicates decisions that have not been cited in these time frames. The increase after ten years in the number of cases cited is relatively limited (2.7%). However, as a ten-year time span would result in a very small training set (as only cases could be selected that were published more than ten years ago), we opted for the five-year time span instead. The majority of cases which get cited in the total time since they were published also get cited in the first five years (on average across the three datasets: 84.7%). Because we forecast the number of citations for a period of five years, we exclude all cases not published at least five years ago (i.e. those published after September 1st, 2017).

We train a model by providing it with the text of the decisions and whether a decision was cited or not (i.e. the ‘labels’). The labels cannot be derived from the texts of the decisions. The model then learns what characteristics (i.e. the ‘features’) are indicative of each label. A held-out development set is used to determine the best algorithm and settings of the algorithm. After the training and development phase, we test the model on data which was excluded from this phase. The selected model thus has to apply the knowledge it has gathered during its training phase to forecast the labels of these new data. We train our model on decisions that are older than the decisions that we test on, which mirrors a real-life situation.

For our experiments, we use the oldest 60% of the data as training data. From the remaining 40%, we use the oldest half (i.e. 20%) as the development data, and the most recent half (i.e. 20%) as the test data. For the final experiment, we build the model including both the training and development data, which means we train on 80% of all the data and test on the remaining 20%. This is a common split used in machine learning, which has empirically been shown to be the best division of train and test data (Gholamy et al. 2018). The respective sizes of the datasets can be found in Table 1. The column called ‘Label’ refers to the value we are forecasting: a 0 label means that a decision received zero incoming citations, a 1 label means a decision received one or more incoming citations.

As Table 1 shows, the data is (sometimes heavily) skewed towards not being cited. To counteract this, we ran some initial experiments with weights assigned to each class in the classifier. However, the performance of the Council of State and the district courts models was very poor, with the model only predicting the label that was present more often (the uncited decisions). We therefore balanced all the training data by undersampling the majority class (for all types of cases). This means that we randomly removed decisions from the majority class (‘uncited’) until it had the same size as the minority class (‘cited’). Table 2 shows the resulting counts per dataset. We did not balance the development and test data, to still simulate a real-life scenario. Note that when adding the development set to the training set for the testing phase, the majority class was again undersampled to ensure that our training data remained balanced.

**Table 1:** Sizes of datasets per court prior to balancing of the training data

Court	Label	Training	Development	Test	Total
Supreme Court	0	9015	3511	3312	15,838
	1	8388	2291	2490	13,169
Council of State	0	28,409	8391	7405	44,205
	1	7204	3480	4467	15,151
District courts	0	86,340	28,358	27,668	142,366
	1	5901	2389	3079	11,369

**Table 2:** Sizes of datasets per court after balancing of the training data

Court	Label	Training	Development	Test	Total
Supreme Court	0	8388	3511	3312	15,211
	1	8388	2291	2490	13,169
Council of State	0	7204	8391	7405	23,000
	1	7204	3480	4467	15,151
District courts	0	5901	28,358	27,668	36,427
	1	5901	2389	3079	11,369

Table 3 shows the sources of the incoming citations for each type of court in our balanced dataset. This table reveals some insights into what our labels consist of, and into the different citation approaches per court. The Supreme Court can be cited by all levels of courts from all areas of law, including itself. The district court can in theory be cited by any court from any law area. In practise, however, district courts tend to mostly only get cited by other district courts. The Council of State only decides in the administrative law area, in which there is no court of appeal. We see these facts reflected in Table 3. Over 96% of the incoming citations of the Council of State originate from the Council itself, and from the district courts. The Supreme Court is cited by all previous levels of courts (e.g. Courts of appeal and district courts). The district courts are mostly cited by themselves.

**Table 3:** Distribution of the incoming citations per dataset

	Citations originating from court (%)							
Cited Court (no. of incoming cit.)	SC	CS	DC	CA	TIAT	CAT	PGO	Other
Supreme Court (143,665)	6.0	0.3	28.0	24.9	0.3	0.8	38.0	1.5
Council of State (58,810)	0.2	52.3	44.1	0.8	0.6	0.8	1.1	0.2
District courts (18,754)	1.6	5.4	56.0	13.0	1.8	5.1	15.9	1.2

*SC* Supreme Court, *CS* Council of State, *DC* district courts, *CA* court of appeal, *TIAT* Trade and Industry Appeals Tribunal, *CAT* Central Appeals Tribunal, *PGO* Procurator General’s Office

### Features

We have extracted a number of features from the available metadata of our datasets. We found 29 different variables, but we only used a selection, since not all of them contribute any valuable information that could help in forecasting the citations. Specifically, we did not use any fixed values (e.g., ‘*language*’, which was always ‘Dutch’), and unique values (e.g., ‘*identifier*’, which is different for every decision used by courts internally), elements that were absent for the majority of decisions (e.g., ‘*temporal*’, indicating if the decision of a case is dependent on a specific time frame), and elements containing information that was not available at the moment of publication of the decisions (e.g., ‘hasVersion’, which contains journals where a decision was later published), and therefore should not be used to forecast citations of decisions. After eliminating these metadata, we were left with the ‘procedure’, ‘law area’, and ‘outgoing citations’, which we transformed as described in the rest of this section.

The outgoing citations contained both citations to case law and legislation, so we split these into citations to domestic law, domestic peak level court decisions, domestic non-peak court level decisions, formal relations that were published earlier in the same case, formal relations with the General-Prosecutor’s Office, and EU case law and legislation. The identifier of the latter did not allow us to easily differentiate between legislation and case law, and thus had to remain combined. As the forms of all of these citations vary substantially, we chose to focus on the number of citations. More information on these types of citations can be found in Table 4.

Furthermore we crafted a number of features based of the work of Van Opijnen (2012). The endogenous features that he used to predict case law importance are the following: type of court, number of judges (which, among other things, reflects the importance or complexity of the case being tried), news item (whether a decision is published on the homepage of rechtspraak.nl), length of the decision, references to European law and domestic law and case law, and the Marc Out-degree (a network analysis algorithm developed by Van Opijnen (2012), based on outgoing citations to Dutch case law). We could not include the Marc Out-degree and the news item features, as this information was not available in our datasets, but all other features were included to a certain extent. The number of judges was included in the previously mentioned ‘procedure’ feature, as this information is sometimes recorded in this line of metadata. We did not use this as a separate feature, as that would require processing each decision to find the number of judges. Instead we chose to just use the information if it was available in the metadata. EU-connotations and cited legislation were present in the previously mentioned citation features. Additionally, we used the text of the summary and the text of the entire decision in the form of n-grams (i.e. sequences of 1 or more consecutive words) as feature sets. The complete list of feature categories that were used in our experiments is shown in Table 4.

**Table 4:** A description of all of the individual features used further along in this paper

Feature name	Meaning
Count_cit_out_peak	The number of unique outgoing citations to decisions by any of the peak level courts of the Netherlands.^a^
Count_cit_out_not_peak	The number of unique outgoing citations to any Dutch court that is not included in Count_cit_out_peak
Count_european_law	The number of unique outgoing citations to CELEX documents. These are documents that can be found on EUR-Lex, and include EU legislation, case law, and other legal documents coming mostly from EU institutions, but also from EU member states, European Free Trade Associations, etc.^b^
Count_domestic_law	The number of unique outgoing citations to Dutch legislation
Count_formal_phr	The number of formal references to advices from the Prosecutor General’s Office at the Supreme Court. Only used for the Supreme Court experiments, as this is the only court that receives these advices
Count_formal_not_phr	The number of outgoing citations to any formal relations published prior to the decision, e.g., decisions by lower or higher courts in the same case this decision is a continuation of
Law_area	The area of law that the decision is made in. Only used for the Supreme Court and the district courts.^c^
Len_decision	The number of words in the text of a decision
Len_summary	The number of words in the text of a summary
Procedure	The type of procedure of the decision. This variable varies per court, but can include whether or not a case is judged by multiple judges, whether or not a decision is an appeal, or if it is a preliminary relief proceeding.^c^
Text_decision	The text of the decision
Text_summary	The text of the summary of the decision

^a^ The peak level courts of the Netherlands are the Supreme Court, the Council of State, the Central Appeals Tribunal, and the Trade and Industry Appeals Tribunal

^b^ A more in depth description can be found at https://eur-lex.europa.eu/content/tools/TableOfSectors/types_of_documents_in_eurlex.html

^c^ All possible values are described at https://data.rechtspraak.nl/Uitspraken

### Feature representation

Not all features in Table 4 are machine-readable. To present the data in a suitable format for the machine learning algorithm, we needed to convert them to a numerical representation. The procedure and law area were ‘one hot encoded’. This means that all values of a categorical feature (such as a law area, with possible options ‘administrative law’, ‘criminal law’, and ‘private law’) are transformed into their own column (law_area_administrative_law’, ‘law_area_criminal_law’, and ‘law_area_private_law’) with a value of either 0 or 1. Originally, the law_area values were divided into some very specific areas, such as ‘private law; law of obligations’. We only preserved the broader law area named before the semicolon, as the latter part was often too specific to be a representative feature as it occurred only very infrequently. For the Council of State, this feature was irrelevant, as all of the decisions belong to the law area of administrative law. In the Supreme Court dataset, 35.3% belongs to administrative law (limited to tax law), 34.6% to criminal law, and 30.2% to private law. In the district courts data, 34.3% is administrative law, 26.0% is criminal law, and 39.6% consists of private law decisions.^9^

In the procedure feature, some values were grouped together for the same reason. Some of the values had to be grouped together into an ‘other procedure’ value, as they were much less frequent other more prevalent values. All groupings are shown in Table 5.

**Table 5:** Description of types of procedures that were grouped together. If the column ‘Contains’ is empty, the value shown in the column ‘Grouped Values’ was used by itself, as it made up a very large percentage of the total

Dataset	Grouped values	Contains	Percentage
Supreme Court	Cassation	Cassation; Cassation in the interest of the law	85.4
	Other procedure	All other values	14.6
Council of State	Preliminary injunction	Preliminary injunction; Preliminary injunction + proceedings on the merits	12.9
	First instance— single judge		11.7
	First instance— multiple judges		17.6
	Appeal		55.4
	Other procedure	All other values	2.4
District courts	First instance— single judge		37.9
	First instance— multiple judges		37.2
	Other procedure	All other values	24.9

For the summary of contents and the complete text of the decision, we used the TfidfVectorizer.^10^ This method converts texts into series of numbers (i.e. vectors) and assigns higher (*tf-idf*) scores to more frequent words which are also more characteristic for a document. Specifically, *tf-idf* is the product of the *term frequency*, which is the number of times a word appears in a document, and the *inverse document frequency*, which is the logarithm of the number of documents divided by the number of documents containing the term. This means that so-called stop words (the most common words in a language) that are present in most, if not all, documents have lower scores than scarce words that are more informative about a document. A more extensive explanation of tf-idf can be found in Medvedeva et al. (2020). It should be noted that the use of tf-idf is a relatively simple approach, and there there have been significant improvements in NLP that have expanded the range of possible techniques to represent features. Examples include word embeddings, neural networks, and transfer learning. In order to establish our baseline models, we have opted for a more basic approach, however.

TfidfVectorizer has a number of parameters that may influence performance, such as removing capital letters or using n-grams (a sequence of multiple words or characters) instead of only focusing on single words or characters. We chose to use word n-grams instead of character n-grams, as we want the results to be human-readable to interpret. We included (1,4) n-grams, which means that sequences of either 1, 2, 3 or 4 words were included as features (i.e., their value being the *tf-idf* score). We did not remove stop words, as using the *tf-idf* already compensates for this.

## Method

### Algorithms

A Support Vector Machine (SVM) was used for our experiments (i.e. trying to forecast whether or not the decision is cited). This algorithm allows us to investigate the weights assigned to the features, and thus we can determine which features made the largest contribution to the prediction. The SVM (Vapnik 1999) is a popular algorithm that performs well in legal classification. For a more elaborate explanation of SVMs, the interested reader is referred to Wu et al. (2008) or Medvedeva et al. (2020). Specifically, in this study, we used scikit-learn’s LinearSVC^11^ algorithm.

### Evaluation

We compare the experiments performed with the SVM algorithm using Matthew’s Correlation Coefficient (MCC). We additionally report accuracy scores and the macro $F_1$-score.

The accuracy consists of the percentage of correctly identified decisions. However, the accuracy does not take into account class imbalance. To account for this, we use the macro $F_1$-score, which is the unweighted average of the harmonic mean of precision and recall for both cited and uncited decisions. Precision is the fraction of correctly classified decisions among the classified decisions (i.e. how many decisions that were classified as ‘cited’ are correct). Recall is the fraction of correctly classified decisions among all the decisions with that label (i.e. how many decisions that belong to the ‘cited’-class have been found by the algorithm). Finally, MCC is a robust metric that only yields a high score if the model performs well for all types of predictions to be made (true positives, false negatives, true negatives, and false positives). MCC-scores range from −1 to 1, and it is generally considered a good metric to evaluate model performance, especially for imbalanced datasets, as it takes class prevalence into account (Chicco and Jurman 2020). As Matthew’s Correlation Coefficient is a specific application of Pearson’s Correlation Coefficient for binary cases, we interpret the results a similar way: an absolute MCC of 0.01 to 0.19 is interpreted as no, or a negligible relationship, 0.20 to 0.29 represents a weak relationship, 0.30 to 0.39 a moderate relationship, 0.40 to 0.69 a strong relationship, and any score above 0.70 is considered to indicate a very strong relationship. To gain insights into the performance for each label separately for the final models, we also have a closer look at precision, recall, and confusion matrices.

All metrics mentioned above, except for MCC, range between 0 and 1. MCC can vary between −1 and 1, but only positive values are meaningful in this case. For all metrics, a higher score indicates a better performance.

### Baseline

A baseline serves as a starting point consisting of a simple model to which we compare the performance of more sophisticated models. As the labels in the development and test sets were not balanced (see previous section), a simple (majority class) baseline model could always predict ‘no citations’. This would result in a model that performs reasonably well, being correct in at least 57% of the decisions in the case of the SC experiment, 62% for the CS, and 90% in the case of the DC experiment. However, we also wanted to assess whether our final model improves over a very simple machine learning model. Consequently, our second baseline model used word unigrams (i.e. features consisting of single words) from the text of the decisions, converted to a bag-of-words (BOW) representation. This means that the words were vectorised using CountVectorizer (which simply tracks the frequency of each individual word). Then a LinearSVM was used for classification, with all possible parameters set to their default values. We report the scores for both the majority class and the bag-of-words baselines on the test data in Sect. 5.

### Feature selection

This study aims to identify how useful certain features are for forecasting whether or not a decision will be cited after its publication. Therefore, we perform experiments to identify what type of feature holds the most information. For this purpose, we combine all features from the metadata, and we combine all features that are textual (the summary and the decision). We also looked at the different types of metadata (categorical and numerical). Then we combined all of these features together. These initial experiments were all evaluated on the development data and compared to the BOW baseline model and majority class baseline described above. The best-performing combination was then used in our final model, which was evaluated using the separate held-out test set. Then, we look into the most informative features of the best-performing model to gain insights into the reasons why a decision might be cited or remain uncited. Finally, since a slightly lower-performing but more explainable model might be preferred over a better-performing but opaque model, we compared the performance of highly explainable models (i.e. those only based on handcrafted metadata features) to the performance of the best model.

## Results

### Determining the best configuration

First, we discuss the experiments in which we compare the different types of features: metadata (numerical and categorical) versus textual.

The results of the Supreme Court experiments on development data can be found in Table 6. The features from all metadata together perform almost as well as the textual features, with strong positive MCC scores of 0.53 and 0.58 respectively. As the combination of all metadata and textual features performed the best (strong positive MCC of 0.58 and a 0.01 increase in $F_1$-score over the model using textual features), this combination was also used in the final SVM model.

**Table 6:** Scores for feature combinations for the Supreme Court on the development set

Features	Accuracy	$F_1$-score	MCC
Majority baseline	0.61	0.00	0.00
BOW baseline	0.72	0.69	0.40
Numerical^a^	0.77	0.76	0.53
Categorica^lb^	0.69	0.68	0.37
Textual	**0**.**79**	0.78	**0**.**58**
All Metadata (Numerical + Categorical)	0.78	0.77	0.53
Textual + Metadata	**0**.**79**	**0**.**79**	**0**.**58**

^a^ Numerical features include counts of outgoing citations to peak and non-peak courts, to domestic and European legislation, to previous rulings in the same case, to advices from the Procurator General’s Office, and the length in words of the summary and the decision

^b^ Categorical features include the area of law and the procedure

The best results per column are highlighted in boldface

The overall performance of the Council of State models is worse than the performance of the Supreme Court and can be found in Table 7. Again, the combination of both metadata and textual features performed best (weak positive MCC of 0.27), and thus this combination was used in the final SVM model. Again using only the metadata was not much worse than using the textual features (i.e.  MCCs of 0.24 and 0.26, respectively).

**Table 7:** Scores for feature combinations for the Council of State on the development set

Features	Accuracy	$F_1$-score	MCC
Majority baseline	**0**.**71**	0.00	0.00
BOW baseline	0.56	0.57	0.17
Numerical^a^	0.59	**0**.**58**	0.24
Categorical^b^	0.53	0.52	0.15
Textual	0.53	0.53	0.26
All Metadata (Numerical + Categorical)	0.56	0.56	0.24
Textual + Metadata	0.56	0.56	**0**.**27**

^a^ Numerical features include counts of outgoing citations to peak and non-peak courts, to domestic and European legislation, to previous rulings in the same case, and the length in words of the summary and the decision

^b^ Categorical features consist of only the procedure

The best results per column are highlighted in boldface

The results for the district courts can be found in Table 8. The DC scores were much lower than the previous courts we have evaluated, with performance peaking at a (negligible) positive MCC of 0.15. The feature set based on the text and the metadata of the decision is again the best performing combination. Based on these results, the final SVM model used a combination of all of the metadata and textual features together for the district courts as well.

**Table 8:** Scores for feature combinations for the district courts on the development set

Features	Accuracy	$F_1$-score	MCC
Majority baseline	**0**.**91**	0.00	0.00
BOW baseline	0.57	0.44	0.06
Numerical^a^	0.64	0.48	0.11
Categorical^b^	0.72	**0**.**50**	0.08
Textual	0.50	0.42	**0**.**15**
All Metadata (Numerical + Categorical)	0.65	0.49	0.11
Textual + Metadata	0.58	0.46	**0**.**15**

^a^ Numerical features include counts of outgoing citations to peak and non-peak courts, to domestic and European legislation, to previous rulings in the same case, and the length in words of the summary and the decision

^b^ Categorical features include the area of law and the procedure

The best results per column are highlighted in boldface

For all the courts, the best-performing model included a combination of all features (both textual and metadata). All models showed an improvement over both baseline models in terms of MCC.

### Best model performance

In Table 9, we have listed the scores of the baseline models, which consist of a bag-of-words model and a LinearSVM with unigrams, and the majority baseline. Both were tested on the held out testset as well. Underneath them are the scores of the models using a combination both the textual and metadata features in a LinearSVM. Compared to the Supreme Court (strong positive MCC of 0.60), the other two courts perform much worse, with the Council of State achieving a weak positive MCC of 0.27 and the district courts a negligible MCC of 0.17. Nevertheless, the baselines of both courts were outperformed on the basis of their MCC scores.

**Table 9:** Comparison between the majority class baseline, bag-of-words baseline, and best SVM model, using metadata and textual features, per court

Dataset	Model	Accuracy	$F_1$-score	MCC
Supreme Court	Majority baseline	0.57	0.00	0.00
	BOW baseline	0.72	0.69	0.40
	SVM	**0**.**80**	**0**.**80**	**0**.**60**
Council of State	Majority baseline	**0**.**62**	0.00	0.00
	BOW baseline	0.56	**0**.**57**	0.17
	SVM	0.55	0.54	**0**.**26**
District courts	Majority baseline	**0**.**90**	0.00	0.00
	BOW baseline	0.57	0.44	0.06
	SVM	0.57	**0**.**48**	**0**.**17**

The best results per column for each dataset are highlighted in boldface

For the best performing models, we looked into the precision and recall scores per label (Table 10), and the confusion matrices (Tables 11, 12, and 13). For all three courts, the precision of the uncited decisions is higher than the precision of the cited decisions. The Supreme Court model has similar $F_1$-scores for both labels, only 0.03 apart, and performs a bit better at forecasting the 0 label, with a particularly high precision when forecasting the uncited cases. The Council of State model’s performance shows comparable performance in terms of $F_1$ score for both labels. However, while it is very precise in labelling uncited cases, it fails to detect many of them (i.e. low recall). The opposite is true for the cited cases. It is not very precise in identifying these, but it identifies almost all of them. Most of the Council of State forecasts are false positives: a decision being forecasted as being cited when in reality, it is uncited. The district courts model shows the greatest difference between $F_1$-scores of labels, with the ‘uncited’ label having a score of 0.70 and the ‘cited’ label merely having a score of 0.25. As the confusion matrix shows, it is very precise in identifying uncited cases, but not at all able to identify cited cases precisely.

**Table 10:** Precision, recall, and $F_1$-scores of the best SVM models per court

Dataset	Label	Precision	Recall	$F_1$-score
Supreme Court	0	0.86	0.78	0.81
	1	0.73	0.83	0.78
Council of State	0	0.84	0.34	0.48
	1	0.45	0.89	0.60
District courts	0	0.95	0.55	0.70
	1	0.15	0.73	0.25

**Table 11:** Confusion Matrix of the best Supreme Court SVM model

	Forecast: 0	Forecast: 1
Actual: 0	2570	742
Actual: 1	433	2057

**Table 12:** Confusion Matrix of the best Council of State SVM model

	Forecast: 0	Forecast: 1
Actual: 0	2501	4904
Actual: 1	487	3980

**Table 13:** Confusion Matrix of the best district court SVM model

	Forecast: 0	Forecast: 1
Actual: 0	15,354	12,314
Actual: 1	840	2239

### Analysing the most informative features

**Fig. 4:**
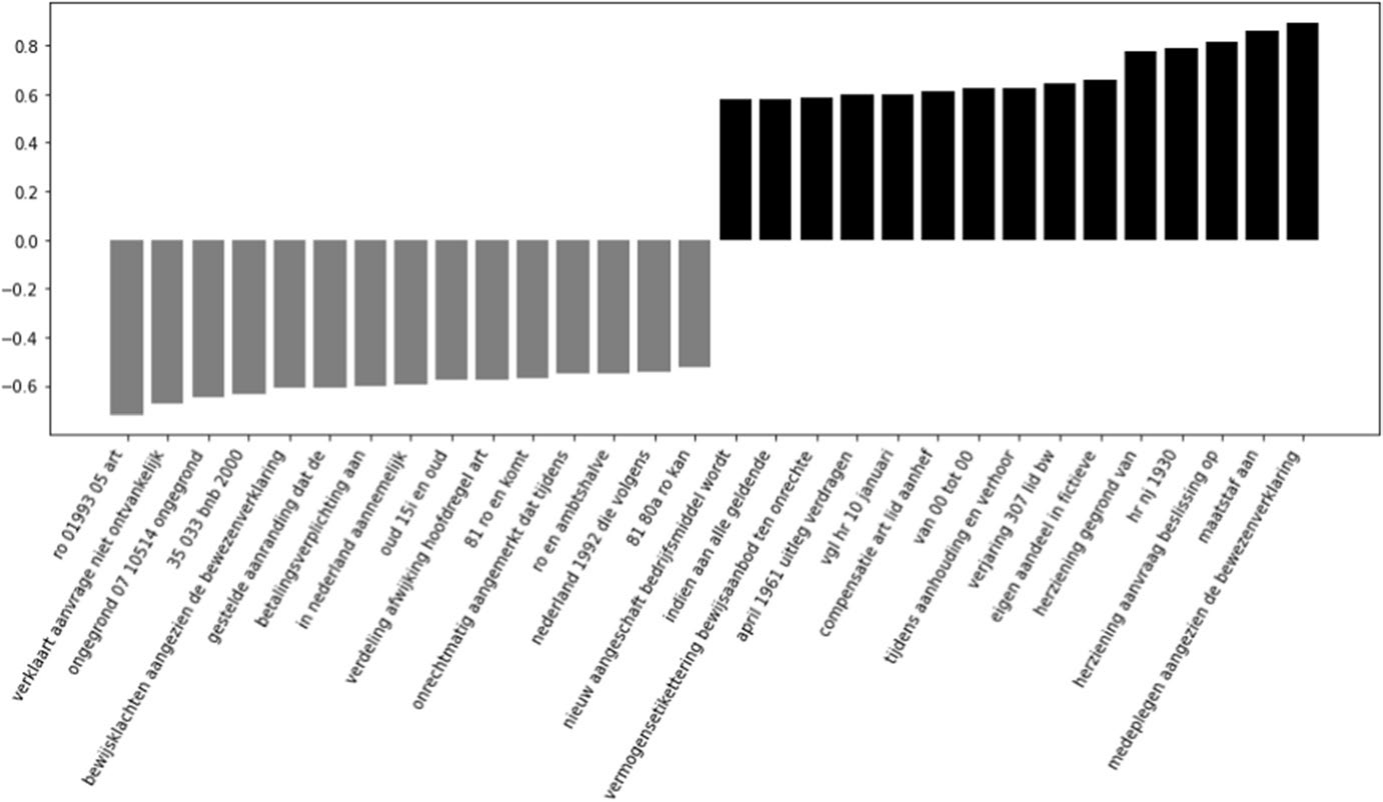
The fifteen most informative features from the summaries of the Supreme Court Decisions per label. Negative scores are informative for the uncited decisions, positive scores are informative for the cited decisions

Because we are interested in the most informative features for a model that works well for both cited and uncited cases, we will investigate the features of the Supreme Court models more in depth. When looking at the most informative features for this model, we only find n-grams that originate from the decisions and summaries. The most informative n-grams from the summaries can be found in Fig. 4. Within the most informative words concerning uncited decisions, we find the words ‘*niet ontvankelijk*’ (inadmissible), and ‘*ongegrond*’ (unfounded). These words are sensible, given that they all indicate decisions that are not ground-breaking. Finally, we also see a number of references to *‘80a’* and *‘81 ro’*, which refers to procedural legislation that the Supreme Court can use to rule on a case without much or even any reasoning. In the most informative summary features for the cited decisions, we find ‘herziening’ (*revision*) and ‘maatstaf’ (*criterion*). These features make sense in light of the work of Fowler and Jeon (2008), who stated that reversed decisions tend to be more important, as well as case law that fills ‘gaps’ in legislation (e.g., by introducing or elaborating on a legal criterion). There is also a term containing *‘hr nj 1930’*, which refers to a specific Dutch legal case law review journal, in which interesting decisions are published with annotations from legal scholars.

**Fig. 5:**
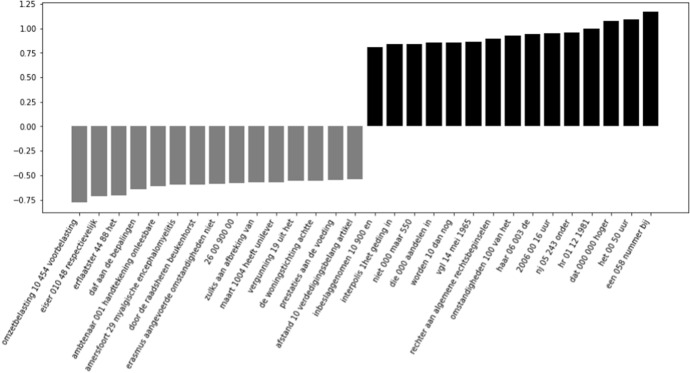
The fifteen most informative features from the decisions of the Supreme Court Decisions. Negative scores are informative for the uncited decisions, positive scores are informative for the cited decisions

In Fig. 5, we find the most informative features that originate from the full text of the decision. On the side of most informative features for uncited decisions, we find a very specific medical feature, *‘myalgische encefalomyelitis’* (chronic fatigue syndrom), and some numbers we assume to be case numbers that the judiciary uses internally. We observe similar numbers for the most informative features for cited decisions, but even legal scholars were not able to decipher what they would be referring to.

For the goal of our research, we prefer false positives (i.e. cases predicted to be cited, but are not) over false negatives (i.e. cases predicted to not be cited, but are in fact cited). We would rather receive a recommendation for a decision that turns out to be irrelevant, than have an important decision filtered out from our results. To find a possible explanation for the false negatives, we manually assessed a randomly selected sample of 10% of the false negative predictions of the best-performing Supreme Court experiment (43 out of 433 documents). We found that all examined decisions, except for one, were very short decisions. Most of them were decisions that were dismissed, deemed unfounded or were inadmissible. 35 out of 43 decisions were ruled without any substantive reasoning, which the Supreme Court is allowed to do according to certain procedural articles which were mentioned before, (*‘artikel 80a R.O.* and *artikel 81 R.O.’*).

### Performance of highly explainable models

N-grams are not completely explainable, which we have seen as not even legal experts could make sense of the most informative n-grams of the best model. This is why we compared the performance of the best-performing model of the previous section to a completely explainable system that uses only metadata features. The results can be found in Tables 14, 15. In line with the development data results, the performance of these models on the test set is similar to that of the best models. The Supreme Court and district courts models perform slightly worse, whereas the Council of State model performs marginally better.

The precision of the uncited decisions is in all instances lower for the metadata-only model, with the largest difference being found for the Supreme Court (0.86 to 0.78). The recall, however, improved for the 0-label in all three cases when using only metadata, with the largest difference being found for the district court model (0.55 to 0.68). The opposite happened for the cited decisions: the precision was better for the models using metadata only (largest difference in the Supreme Court, which went up from 0.73 to 0.79), while the recall went down in all cases (largest differences for the Council f State, which went down from 0.89 to 0.74 and the district courts, which went down from 0.73 to 0.53).

The metadata-only models forecast the uncited-label more often, which is reflected in higher overall accuracy and $F_1$-scores for the Council of State and district courts (see Table 14), which makes sense as for those datasets there are many more uncited than cited cases.

**Table 14:** Comparison between an SVM using metadata features and the best-performing model using textual and metadata features

Dataset	Model	Accuracy	$F_1$-score	MCC
Supreme Court	SVM Meta	0.78	0.77	0.55
	SVM Meta + Text	**0**.**80**	**0**.**80**	**0**.**60**
Council of State	SVM Meta	**0**.**58**	**0**.**58**	**0**.**27**
	SVM Meta + Text	0.55	0.54	0.26
District courts	SVM Meta	**0**.**67**	**0**.**51**	0.13
	SVM Meta + Text	0.57	0.48	**0**.**17**

**Table 15:** Precision, recall, and $F_1$-scores of the SVM models using only metadata per court

Dataset	Label	Precision	Recall	$F\_1$-score
Supreme Court	0	0.78	0.84	0.82
	1	0.79	0.79	0.71
Council of State	0	0.81	0.42	0.56
	1	0.47	0.74	0.60
District courts	0	0.93	0.68	0.79
	1	0.16	0.53	0.24

**Fig. 6:**
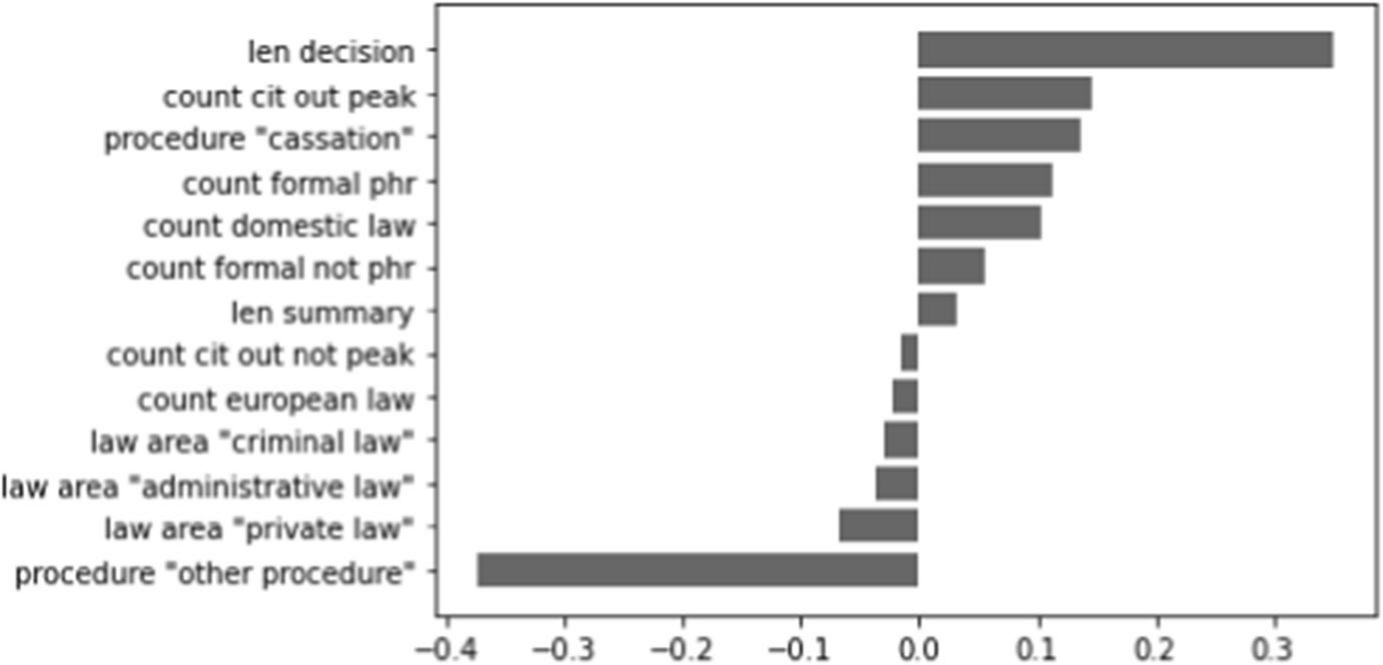
Contribution of metadata features for the Supreme Court

**Fig. 7:**
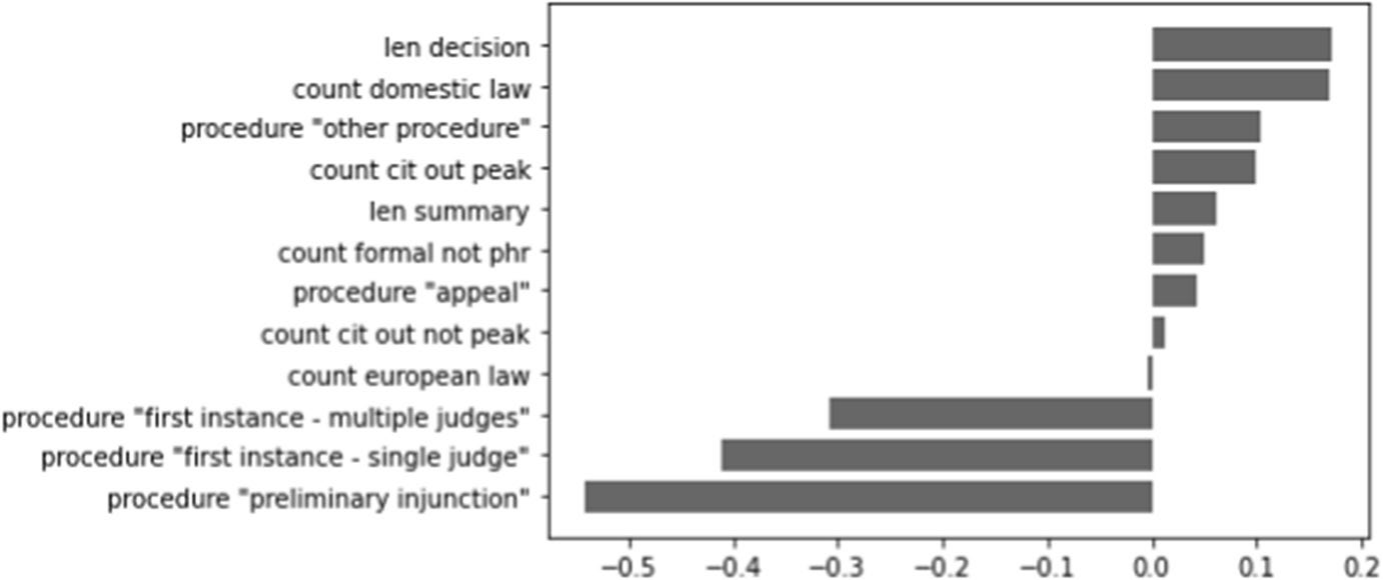
Contribution of metadata features for the Council of State

**Fig. 8:**
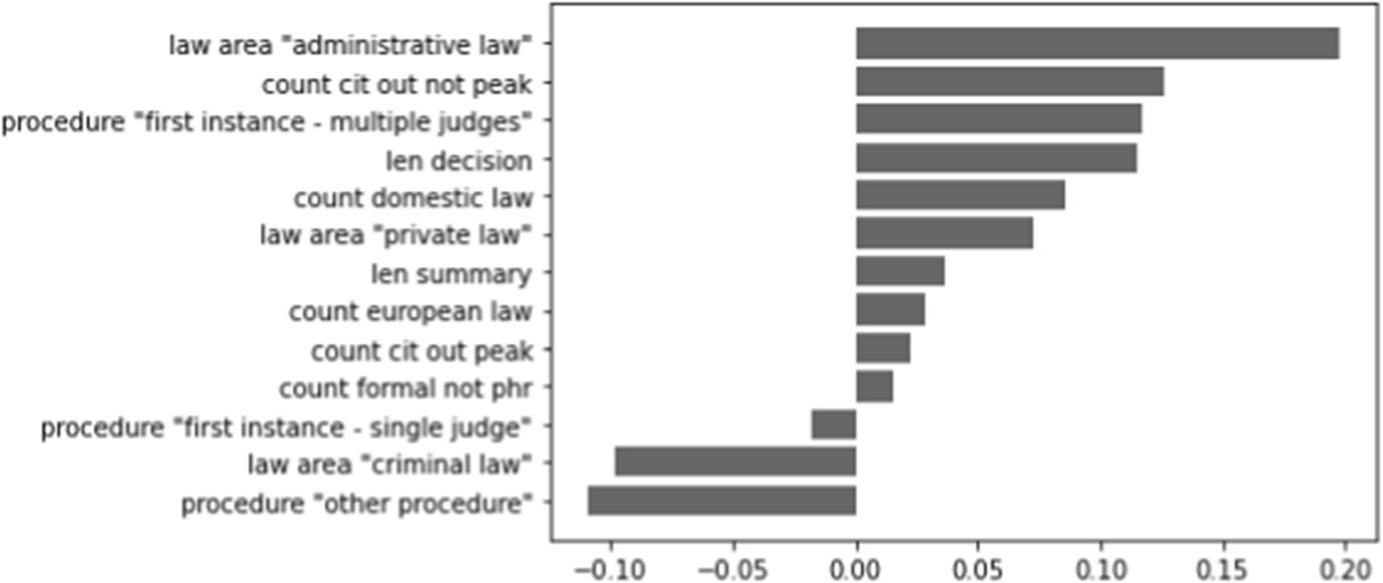
Contribution of metadata features for the district courts

We are interested in which features are contributing most to the performance of the model only including metadata. In Figs. 6, 7, and 8 (shown below), a visualisation of the metadata features is displayed, ranked according to their coefficients. A negative contribution indicates that the feature is useful for forecasting the 0-label, whereas a positive contribution is helpful for forecasting the 1-label.

In Fig. 6, we see that the length of Supreme Court decisions contributes the most out of all metadata features to determine if a decision is cited. The other most indicative feature is ‘other procedure’ (which, in this case for the Supreme Court, distinguishes between cassations and non-cassations), which is more closely related to uncited decisions. The influence of the other features is clearly much less strong. For the Council of State, we mostly see substantial influences on the negative side in Fig. 7: all procedure types except for the ‘other procedure’ are indicators for uncited decisions. The length of the decision and the number of citations to domestic law are again among the top contributors to determine cited cases. In Fig. 8, we find that the (negative) coefficients of the district courts model are smaller than those of the Supreme Court and the Council of State. The largest contribution on the positive side is made by the law area ‘administrative law’, whereas ‘criminal law’ is a contributor on the negative side of the graph. ‘Other procedure’ is again a contributor on the negative side, as is ‘first instance - multiple judges’ being more likely to be associated with a cited case.

## Discussion

In this section, we discuss our main findings and their possible explanations: 1) All SVM models are reasonably well suited to identify whether or not a decision will be cited; 2) When our models predict a decision will not be cited, it is quite precise and can therefore be used to filter decisions; 3) The Supreme Court models outperform the models of the other courts; 4) The simpler metadata models perform almost as well as more complicated models that use the entire judgment, and sometimes even better.

The first finding we discuss is the performance of the SVM models. Our research confirmed that, at least for the Supreme Court models, a linear SVM model is able to predict whether or not a decision will be cited. While the general results were much worse for the Council of State and the district courts’ decisions, for all three courts, the precision of predicting uncited decisions was quite high (ranging between 0.84 and 0.95 for our best models). While the recall of uncited decisions is not very high for the Council of State and the district courts, the decisions it does filter out are likely uncited. This result makes sense, as the uncited-label is a clear-cut class in which all decisions receive zero citations. At the same time, the cited-label is a scale in which all decisions could receive anywhere between one and thousands of citations. Consequently, our second finding is that using our systems for filtering out non-authoritative decisions, i.e. before attempting to identify the most authoritative decisions, is certainly feasible. While this imbalance regarding classes might seem strange, it does make sense, as decisions which are not cited are not authoritative. On the other hand, decisions that are cited may be authoritative (if they are cited often) or non-authoritative (if they are only cited infrequently). Consequently, future work might look at predicting the citation frequency.

Our third finding is that the Supreme Court models outperform the models of the other courts by a large margin. The best Supreme Court model has an MCC of 0.60, which can be considered a strong positive score. However, the MCC score of the best Council of State model is only 0.26 (weak positive), whereas it is negligible (0.17) for the district courts.

A possible explanation for the difference between the Supreme Court and district courts may be the difference in the variety of decisions. District courts are known as ‘fact-finding courts’, focusing on the facts and the evidence of a case, whereas the Supreme Court is a court of cassation. This means that the Supreme Court only assesses some aspects of a case, and it does not, for example, substantively reassess the facts and evidence of a case. Under Dutch law, the Supreme Court only reviews whether a lower court (i.e. district court or court of appeal) applied and interpreted the law (in)correctly and applied the procedural rules properly. Consequently, not all decisions are fit for cassation. The Supreme Court will declare an appeal inadmissible or dismisses the appeal with a short and standard ruling in several situations. Specifically, it will do this if the cassation appeal does not focus on the interpretation of the law, if the Supreme Court already ruled on the interpretation of the contested law in a previous decision, if the court of appeal has sufficiently explained its judgment, or if new facts are presented in the cassation appeal.

District courts do not have such strict prerequisites and rule on a wider variety of decisions than the Supreme Court. The wide variety of district court decisions could result in a greater discrepancy between the test data and the training data of the district courts. For example, a very specific theme in the training data might never be mentioned again in later decisions in the test data, or there could be new topics present in the test data that the model has not seen before in the training data.

While this large variety of decisions explains the low general performance of the district court models, it does not explain the low performance for the Council of State, as the latter’s rulings are limited to administrative law only.

However, a reason for this lower performance may be related to the organisation of the Dutch judiciary. Before a case ends up at the Supreme Court, a district court and a court of appeal decision are made in nearly all private and criminal law decisions. However, administrative law has only two levels of courts: district courts and the Council of State. There are no courts of appeal in administrative law. Consequently, without a court of appeal, there is no ‘filter’ between the court of first instance (district courts) and the court of last instance (the Council of State). In other words, if a party disagrees with the assessment of the facts of the case, the evidence that has been brought forward, or the motivation of the district court, the party could go straight to the Council of State. The lack of a filter, in combination with the fact-finding role of the Council of State, might diminish the authority of their decisions. This could explain why it is much harder to forecast whether or not the Council of State is cited: there is not as much meaning attached to these decisions, which may be reflected in the lack of meaningful words in the text of the decision.

Finally, the difference in performance between the Supreme Court model and the other models could be traced back to the distribution of the data itself. We balanced the training data of all of the courts, but the imbalance in the Supreme Court data was the smallest originally. This means that the SC models were trained on the most true-to-life distribution and the smallest amount of data was lost in the process of balancing. We removed 3.6% of the SC training data, whereas the CS and DC lost 59.5% and 86.3% of their training data, respectively. For future research, we recommend investigating the effects of the data splits further, either by looking into the effects of balancing per court in-depth, or by investigating different train-test-development splits.

For the goal of our research, we prefer false positives (i.e. cases predicted to be cited, but are not) over false negatives (i.e. cases predicted to not be cited, but are in fact cited). We would rather receive a recommendation for a decision that turns out to be irrelevant, than have an important decision filtered out from our results. While the Supreme Court model generally performed the best, it also showed the highest percentage of false negatives in its confusion matrix (see Table 11). For SC, CS, and DC respectively, the percentages of false negatives out of the total forecasts of the best-performing SVM model were 7.5%, 4.1%, and 2.7%. As mentioned before, both the Council of State and district courts are fact-finding courts. The range of subjects that they judge is much wider than the more abstract decisions of the Supreme Court. A large part of the uncited decisions of the CS and DC model was left out of the training data for the purpose of training the model not to predict the majority class, but this means that the model only learnt of a small part of uncited decisions.

In Sect. 5 we have seen that there is an overlap between the most informative features that the model has learnt for uncited decisions, and the characteristics of false negative predictions of the model. Upon further assessment by legal scholars, they qualified the texts of these false negative decisions as non-authoritative. Courts seem to cite these decisions in similar circumstances, and employ these citations to substantiate the omission of their own motivation. Consequently, our model appears to be capable of identifying cited decisions that lack any information for future decisions (i.e. decisions that lack authority). We believe that it would be reasonable for these cited decisions to be excluded by a non-authority filter. One false negative could not be explained by legal scholars, but we need to accept that our model is not flawless.

The fourth and final finding was that the performances of the models utilising only metadata features were reasonably close to the performance of the models using all (including textual) features. It is interesting that an explainable model with far fewer features performs (almost) as well as a less explainable model. Even though we attempted to create an explainable SVM model through the use of word n-grams and no preprocessing of words, legal experts still could not make sense of most informative textual features. Consequently, the lower performance of a simpler model may be preferred if the simpler model is (better) explainable. We should note that we have only used metadata features that could be extracted from the metadata section of the XML files. Further research should be conducted into the extraction of specific metadata features from the text of the decision, either by manual annotation or a reliable extraction algorithm. Examples include the involvement of children, drugs, legal counsel, or the gender of the judge or the parties involved.

Currently, we have only looked into the text of the decision as a whole, but not separate parts of the text. By separating the text into different paragraphs, such as the facts and the reasoning of the court, the most informative parts of the decisions can be further investigated. It is possible that the most informative features will make more sense to legal scholars in that case.

As the scope of our experiments was mostly focused on establishing a baseline in a new task, we have not used the most state-of-the-art NLP techniques (e.g. deep learning or large language models) in our research. This entails that the performance of the metadata models is compared to models that are not the most sophisticated models. In future research, an interesting comparison could be drawn between our explainable metadata-only models and less explainable advanced models based on deep learning. Nevertheless, for the task of identifying non-authoritative cases, our relatively simple machine learning models exhibit impressive performance.

In Sect. 5, we found that, generally speaking, the length of decisions and the outgoing citations to peak courts are large contributors to the performance of the models.

The importance of the citations to peak court decisions could be explained by the difference between the role and function of the peak courts compared to those of other courts. Citations to the Supreme Court are among these peak court citations. As mentioned before, the Supreme Court is a court of cassation under Dutch law. This not only has implications for the aspects of the case that the court reviews, but also for the function of the appeal. In contrast to appeals to other courts, the function of cassation is not only to protect the interests of the plaintiff, but also (and maybe even more so) to ensure legal certainty and uniformity and contribute to the law’s development. If new phenomena occur for which the law offers no ready-made solution (yet), or if new insights arise regarding what is fair and just, the Supreme Court has to provide guidance on how to deal with such developments (Verheugt 2020). This different role and function of the Supreme Court compared to the other courts could explain why citations to peak-level decisions are a useful feature for forecasting the authority of a Supreme Court decision and a decision of the Council of State. When we look at Tables 6 and 7, we see that these references to peak courts are usually self-references, as cross-references rarely happen. The Supreme Court referring to its own decisions might be indicative of a decision’s contribution to the development of the law. In turn, such a decision might be interesting for future referencing as well. This way, a whole network of citations is created. Research on the different functions (i.e. filling a ‘gap’ in legislation, reversing a previous decision, summarising similar situations, etc.) of citations is needed to discover if the Supreme Court itself is the reason that outgoing citations to peak-level courts perform reasonably well as a predictor, and discover why this feature is not working as well for the district courts. Also, other features might be usefully investigated in future work, such as references to specific laws (Van Opijnen 2012) and treaties or to international legislation in particular.

The function of the Supreme Court could also explain why the length of the decision is a large contributor to the SC model. An important function of supreme courts is to fill ‘gaps’ in legislation (Fowler and Jeon 2008). As such, Supreme Court decisions could provide new interpretations of existing law or offer solutions to problems for which no law yet exists. Evidently, such decisions need more explanation than decisions in which the Supreme Court dismisses an appeal. In contrast to the other courts, the Supreme Court could, for example, dismiss a decision on procedural grounds without explaining (see Articles 80 and 81a of the Dutch Judicial Organisation Act). As such, the function of the Supreme Court might also explain why the length of the decision is such a good indicator.

The fact that judgments can become redundant over time is overlooked by using citations as a proxy for authority. This redundancy could be accounted for by a separate filter that has access to the actual citations within the Dutch judiciary, which might incorporate a reverse function of the Sleeping Beauty coefficient (Hernandez Serrano et al. 2020). Since our present research is focused on predicting non-authority rather than authority, this is less of an issue, but this factor should definitely be accounted for in case of an importance filter.

Finally, we should note that a particular selection bias is in place. Until 2012, the publication of court decisions was based on qualitative criteria such as media attention, the importance for public life, and consequences for the application of regulations. As of 2012, decisions of all peak courts (e.g., the Supreme Court and Council of State) should always be published unless the decision is “unfounded or inadmissible and/or dismissed with a standard reasoning”.^12^. Decisions from the district court should be published if a case received attention from the media or if the decision was of importance for further rulings. These criteria do not limit courts, as they can develop additional criteria or decide to publish every decision they make. Yet, these publication guidelines imply a certain selection bias on rechtspraak.nl towards more authoritative decisions. Once the judiciary starts publishing the vast majority of its case law, it is likely that rechtspraak.nl will contain relatively fewer decisions by the district courts that are cited, as the ‘unimportant’ decisions that currently remain unpublished will be published as well. This reduction in the cited-to-uncited ratio could complicate the process of creating a well-performing model that will also perform well in the future.

## Conclusion

In this study, we have found that the text and metadata of decisions hold information that can be used to forecast whether or not a case is cited.

While our current models are not accurate enough to provide a sufficient prediction for all different types of courts regarding both labels, our systems can be used as a first filter. While the predictions about being cited are not very trustworthy for the Council of State and district court decisions, for all three courts predictions indicating the decisions that will not be cited are fairly reliable, with the district courts model showing a precision of 0.95 when predicting uncited decisions. This means that when our systems indicate a decision is not authoritative (i.e. it won’t be cited), it is likely correct and priority could be given to other decisions to save time. Our study also serves as a first baseline in an experiment that has not been carried out before. Particularly, the experiments regarding the Supreme Court of the Netherlands have been very promising, yielding accuracy and $F_1$-scores of 0.80, and a strong positive MCC score of 0.60.

Finally, our results showed that a simpler, more explainable model using only a dozen features, did not perform much worse than a model using millions of textual features. It is worthwhile to investigate the tradeoff between explainability and performance in future work.

## Footnotes

1 https://jaarverslagrechtspraak.nl/h3-cijfers

2 https://www.nrc.nl/nieuws/2021/05/30/baas-rechtspraak-de-rechtsstaat-staat-weer-op-de-agenda-a4045430

3 https://www.ECtHR.coe.int/Documents/HUDOC_FAQ_ENG.pdf

4 https://www.rechtspraak.nl/Uitspraken/paginas/open-data.aspx, accessed on 27-09-2022.

5 https://www.rechtspraak.nl/SiteCollectionDocuments/Technische-documentatie-Open-Data-van-de-Rechtspraak.pdf

6 https://data.overheid.nl/community/application/1536/datasets, accessed on 23-09-2022.

7 ECLI:NL:HR:1905:1.

8 Application no. 38773/97.

9 Due to rounding of numbers, the total is not exactly 100%.

10 https://scikit-learn.org/stable/modules/generated/sklearn.feature_extraction.text.TfidfVectorizer.html

11 https://scikit-learn.org/stable/modules/generated/sklearn.svm.LinearSVC.html

12 https://www.rechtspraak.nl/Uitspraken/paginas/selectiecriteria.aspx
